# Assessment of *Rhizobium anhuiense* Bacteria as a Potential Biocatalyst for Microbial Biofuel Cell Design

**DOI:** 10.3390/bios13010066

**Published:** 2022-12-31

**Authors:** Viktorija Reinikovaite, Sarunas Zukauskas, Rokas Zalneravicius, Vilma Ratautaite, Simonas Ramanavicius, Vytautas Bucinskas, Monika Vilkiene, Arunas Ramanavicius, Urte Samukaite-Bubniene

**Affiliations:** 1 Department of Physical Chemistry, Institute of Chemistry, Faculty of Chemistry and Geosciences, Vilnius University, Naugarduko Str. 24, LT-03225 Vilnius, Lithuania; 2 Department of Nanotechnology, Centre for Physical Sciences and Technology, Saulėtekio Av. 3, LT-10257 Vilnius, Lithuania; 3 Department of Electrochemical Material Science, Centre for Physical Sciences and Technology, Saulėtekio Av. 3, LT-10257 Vilnius, Lithuania; 4 Department of Mechatronics, Robotics, and Digital Manufacturing, Faculty of Mechanics, Vilnius Gediminas Technical University, J. Basanaviciaus Str. 28, LT-03224 Vilnius, Lithuania; 5 Lithuanian Research Center for Agriculture and Forestry, Instituto Ave. 1, Akademija, LT-58344 Kėdainiai, Lithuania

**Keywords:** *Rhizobium anhuiense* bacteria, electrocatalysis, microbial fuel cell, power density, charge transfer, bioelectrochemistry, bioelectronics, menadione, redox mediator, bio-anode

## Abstract

The development of microbial fuel cells based on electro-catalytic processes is among the novel topics, which are recently emerging in the sustainable development of energetic systems. Microbial fuel cells have emerged as unique biocatalytic systems, which transform the chemical energy accumulated in renewable organic fuels and at the same time reduce pollution from hazardous organic compounds. However, not all microorganisms involved in metabolic/catalytic processes generate sufficient redox potential. In this research, we have assessed the applicability of the microorganism *Rhizobium anhuiense* as a catalyst suitable for the design of microbial fuel cells. To improve the charge transfer, several redox mediators were tested, namely menadione, riboflavin, and 9,10-phenanthrenequinone (PQ). The best performance was determined for a *Rhizobium anhuiense*-based bio-anode mediated by menadione with a 0.385 mV open circuit potential and 5.5 μW/cm^2^ maximal power density at 0.35 mV, which generated 50 μA/cm^2^ anode current at the same potential.

## 1. Introduction

Biofuel harvesting is emerging as an alternative solution in energetics to conventional batteries and holds great potential to design self-powered autonomous low-power electronic devices such as sensors and biosensors. The high demand for ’green-renewable‘ energy has led to a surge of research into various biofuel cell (BFC) concepts [1,2]. BFC-based technologies have the potential to transform the chemical energy of organic waste directly into ‘bioelectricity’ avoiding direct fuel combustion [3]. Microbial fuel cells (MFC) are bio-electrochemical devices that are driven by the metabolic activities of microorganisms [4,5]. The microorganisms can be used in the development of environmentally friendly energy generation systems. There is a great possibility to integrate BFCs within biosensing devices [6]. However, mostly the voltage generated by individual MFCs is too low to power electronic devices [7], and for this reason, they are connected in serial mode [8]. Some MFCs can be integrated into advanced power management systems [9,10]. In order to increase the power of BFC flow-through systems, they can be designed [11]. The anodic compartment of most BFCs is powered by organics [12], where the versatile catalytic properties of microorganisms can be well exploited [13]. The applicability of microorganisms in MFC design is reported in numerous references [5,14]. The ‘electroactivity’ of microorganisms can be improved by *c*-type cytochromes (cyt *c*) [15], type IV pili/nanowire [16], chemically incorporated conducting polymers [17,18], and redox mediators [19,20]. Most MFCs are based on (i) mediated or (ii) direct charge transfer [21]. *Shewanella putrefaciens* [22], *Geobacter sulfurreducens*, *Geobacter metallicreducens* [23], *Aeromonas hydrophila* [24], and *Rhodoferax ferrireducens* [25] are frequently used in direct charge transfer based MFCs. In mediated electron transfer-based MFCs, redox mediators such as methylene blue [26], menadione [27,28], and riboflavin [29] are frequently used to increase the rate of fuel conversion into electricity [4,30]. Diffusion rates of organic fuel molecules can significantly limit MFCs power [31]. Therefore, sometimes it is reasonable to apply various mixing approaches [11]. The negatively charged phosphate and carboxyl groups, which are involved in the cell wall of Gram-negative bacteria, outweigh the positively charged amino groups. Therefore, such conditions determine the rather low surface zeta potential of the mentioned microbes [32], which is important for the interaction of cells with electrodes and consequently for charge transfer.

*Rhizobia* are among the best-studied soil microbes associated with plants [33]. *Rhizobia* bacteria exhibit an oligotrophic lifestyle, belonging to either α-proteobacteria or β-proteobacteria. The interaction is reciprocal where plants selectively enrich beneficial bacteria attaching to their roots, and the *Rhizobia* bacteria infect and colonize plant epidermis, form bacteroids, and then start to fix atmospheric nitrogen; this process is essential for the development of plants. In most agroecosystems, *Rhizobia* are one of the common soil bacteria, especially if legumes are incorporated into the rotation [33]. *Rhizobia*-related bacteria are Gram-negative nitrogen-fixing bacteria that are found throughout Eurasia in an endosymbiotic relationship with legume plant nodules. *Rhizobia*-related species could serve as good candidates for MFC design because they are a non-pathogenic, facultative anaerobe group of microbes. Moreover, *Rhizobium* species have wide capacities to decompose organic substrates, root exudates including C4 dicarboxylates (malate, fumarate, and succinate) and some other short-chain fatty acids. In the native habitat, most of the extracted electrons are usually channeled to nitrogenase to fix atmospheric nitrogen to ammonia. It is used by legume hosts, and a small amount is released into the soil. Dicarboxylate decomposition and nitrogen fixation processes are tightly regulated by metabolic processes performed within the hosting plant. According to scientific research, it is well known that *Rhizobia* species are well-equipped with highly efficient metabolic systems capable of extracting electrons from organic compounds [34]. It should be noted that in laboratory conditions, in the absence of a specific host and conditions in the surrounding environment, these regulatory signals, which are inducing nitrogen fixation, will be absent, and all captured electrons will increase the reduction potential of the *Rhizobia* bacteria-based system. All the aforementioned traits indicate that microbes, which interact with plant roots in the rhizosphere, can be used to develop a new design of microbial fuel cells, and *Rhizobium bacteria* are among the most attractive candidates for this purpose [35].

In this study, we assessed *Rhizobium anhuiense* as a potential candidate for microbial fuel cell design. To improve the charge transfer, we have assessed the *Rhizobium anhuiense*-based bioanode with several redox mediators (menadione, riboflavin, and 9,10-phenanthrenequinone (PQ)) and determined the open circuit potential, maximal power density, and some other electrochemical and electrical characteristics of the here designed bioanodes of MFCs.

## 2. Materials and Methods

### 2.1. Materials

Agar, yeast cell extract, dipotassium hydrogen phosphate (K_2_HPO_4_), magnesium sulfate (MgSO_4_), ferrous sulfate (FeSO_4_), calcium carbonate (CaCO_3_), sodium chloride (NaCl), and D-(+)-glucose (C_6_H_12_O_6_) were purchased from Carl Roth GmbH & Co. (Karlsruhe, Germany). Both 2-methyl-1,4-naphthoquinone (C_11_H_8_O_2_) and riboflavin (C_17_H_20_N_4_O_6_) were purchased from Alfa Aesar (Haverhill, MA, USA). Sodium molybdate (Na_2_MoO_4_) and 9,10-phenanthrenequinone (C_14_H_8_O_2_) were purchased from Merck (Darmstadt, Germany). Yeast cell extract and glucose were of microbiological grade. The other chemicals were GPR-grade.

### 2.2. Cultivation and Preparation of Rhizobium anhuiense Bacteria

Gram-negative *Rhizobium anhuiense* bacteria were obtained from the Lithuanian Research Center for Agriculture and Forestry (Vėžaičiai, Lithuania) microbial strain collection. Bacteria were cultivated in Norris medium [36], pH 7.0, composed of nitrogen-fixing bacteria strains. Prior to use in experiments, the *Rhizobium ahnuiense* bacteria were reinoculated on an inclined Norris medium supplemented with agar and left to grow for 48 h at 28 °C. Afterwards, 5 μL of an autoclaved Norris medium solution was used to fill the test tube with inoculums and carefully suspend it. The mixture was vortexed for several minutes to ensure that the majority of the bacteria got lifted from the solid medium. Then, the bacterial suspension was transferred and diluted in a fresh autoclaved Norris medium to obtain a density of colony-forming units (CFU) equal to 1 × 10^7^ CFU mL^−1^ [37]. The bacteria count was established by measuring the optical density of the suspension at 600 nm (OD_600_), which was measured to be in a range of 0.15–0.2, which corresponds to ~2 × 10^7^ CFU mL^−1^. The bacterial suspension was left to grow for 24 h at 30 °C with constant shaking at 200 RPM to reach the stationary phase, where the OD_600_ was about 1.0. The optical density (OD) of the bacterial culture at a 600 nm wavelength was measured with a spectrophotometer (Ocean Optics USB4000, Largo, FL, USA). Before the experiments, the cells were adequately washed from the broth culture three times with 0.1 M PBS, pH 7.0, and centrifuged at 4500 rpm for 5 min at 25 °C. For all investigations, washed cells were resuspended in the test solution to prepare bacterial suspensions containing ~2 × 10^7^ CFU mL^−1^.

### 2.3. Assessment of Zeta Potential

The prepared samples were diluted 10-fold in a medium with appropriate ionic strength (0.01 mM, 0.05 mM, 0.1 mM, 0.3 mM, 10 mM, 50 mM, 100 mM, and 300 mM), and a pH of 5.0, 6.0, 7.0, or 8.0 was adjusted immediately prior to zeta potential measurement. The resulting suspension was used to fill clear, disposable ‘zeta cells’ (ATA Scientific, Caringbah, Australia) immediately prior to zeta potential measurements. The ionic strength was measured at pH 7.0, while pH investigations were conducted in a 0.1 mM PBS solution. The electrophoretic mobility of bacterial cells was measured with a zeta potential analyzer at 80 V (Zetasizer Nano series Nano-ZS; Malvern Industries Ltd., Malvern, UK) and converted to zeta potentials [38]. Measurements were performed at 25 °C in standard Norris medium pH 7.0. The sample was measured five times on two separate days to determine the reproducibility of the results. Between each measurement, the electrodes were rinsed with copious amounts of Milli-Q^TM^ water, followed by the test bacterial suspension.

### 2.4. Electrochemical Assessment of Microbial Biofuel Cell

A single chamber MFC cell was assembled in which a two-electrode electrochemical system was utilized, where a graphite electrode was used as a working electrode and a large-area platinum wire of 0.5 cm^2^ served as a counter electrode. Bacterial samples were immobilized on a graphite electrode by letting them lightly dry for two minutes. A polycarbonate membrane with 1 μm holes was used to separate the specimen from the surrounding environment to ensure attachment to the anode. The chamber was filled with 0.1 M PBS, pH 7.0, and the open current potential (OCP) was measured. Afterwards, various external resistors (10 MΩ, 1 MΩ, 390 kΩ, 220 kΩ, 180 kΩ, 130 kΩ, 100 kΩ, 68 kΩ, 56 kΩ, 33 kΩ, 10 kΩ, and 1 kΩ) were connected in parallel to the electrical circuit to imitate an external load and to assess the power density of the bacterial samples. The potential changes were recorded with a digital benchtop multimeter (UT8802E UNI-T) from TEM Electronic Components (Łódź, Poland). The power density was calculated by dividing electric power, which was calculated using Ohm’s law, per exposed working electrode surface of 0.07 cm^2^.

## 3. Results and Discussion

**Figure 1:**
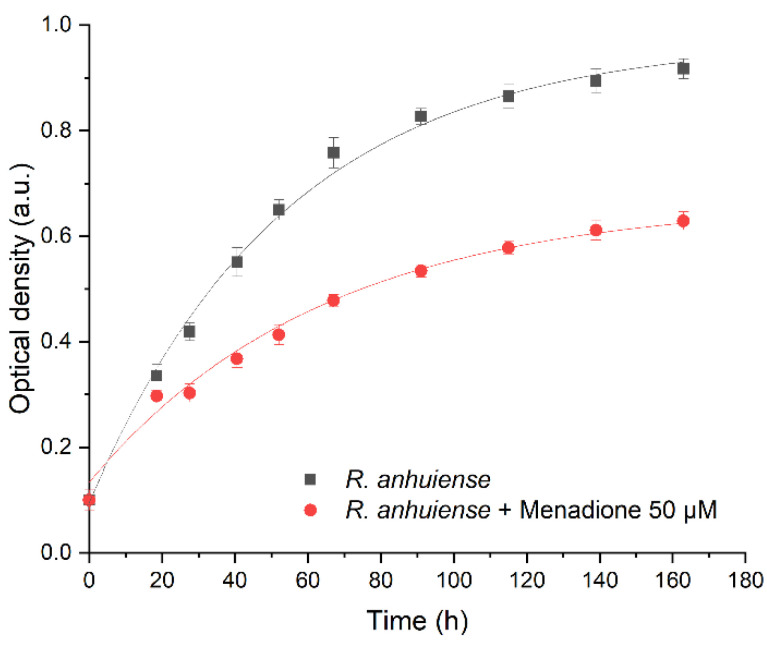
Growth curve optical density (OD) values of (•) *Rhizobium anhuiense* bacteria and (▪) *Rhizobium anhuiense* bacteria with 50 μM of menadione in Norris medium. Data approximated according to exponential function $y=y_{0}+A_{1}e^{-\left(x-x_{0}\right)/t_{1}}$, where: (i) *y*_0_ = 0.97, *x*_0_ = 0.56, *A*_1_ = −0.87, and *t*_1_ = 54 for *Rhizobium anhuiense* bacteria; (ii) *y*_0_ = 0.67, *x*_0_ = 3.28, *A*_1_ = −0.51, and *t*_1_ = 64 for *Rhizobium anhuiense* bacteria with 50 μM of menadione.

The growth of *Rhizobium anhuiense* bacteria in the presence of 50 μM of menadione was evaluated by the determination of optical density after different time periods (Figure 1). An exponential function was applied for the assessment of cell growth:

(1) $$y=y_{0}+A_{1}e^{-\left(x-x_{0}\right)/t_{1}}.$$

The increase in *Rhizobium anhuiense* bacteria number was calculated according to Equation (2):

*Y* = *y*_0 (in the absence of menadione)_/*y*_0 (in the presence of menadione)_. (2)

The time interval during which the cell number has been doubled was calculated according to Equation (3):

*T* = *t*_1 (in the absence of menadione)_/*t*_1 (in the presence of menadione)_. (3)

Data presented in Figure 1 illustrate that the increase of *Rhizobium anhuiense* bacteria number (*Y*), which was calculated using Equation (2), was 40% faster in the case if menadione was absent in the cell growth media, but the time interval during which cell number has been doubled, which was calculated using Equation (3), was 20% shorter for *Rhizobium anhuiense* bacteria that were growing in the presence of 50 μM of menadione.

Zeta potential measurements were performed to evaluate the electrical potential of the cell surface, which depends on environmental conditions [33]. The surface of the majority of Gram-negative bacteria at neutral pH is mostly negatively charged. This effect is predetermined by negatively charged phosphate and carboxylate groups containing lipopolysaccharides [34] and balanced by oppositely charged counterions present in the surrounding media, leading to the formation of the electrical double layer.

**Figure 2:**
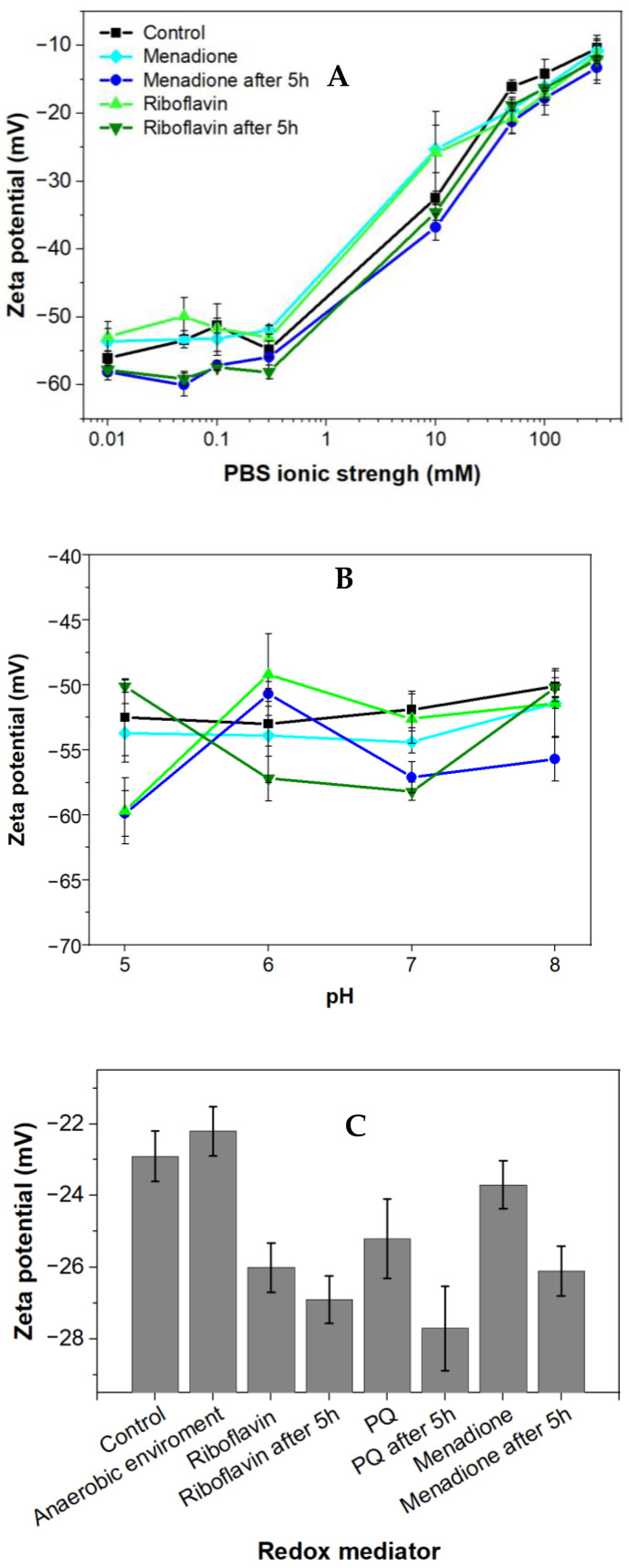
Zeta potential (mean ± SD) of *Rhizobium anhuiense* at mid-logarithmic phase: (**A**) Zeta potential as a function of ionic strength; (**B**) zeta potential measured in 0.1 mM PBS as a function of pH; and (**C**) zeta potential values in growing Norris medium at pH 7.0. Measurements were performed in PBS at pH 7.0 in the presence of several redox mediators (5 mM of menadione, 0.2 μM of riboflavin, and 4 mM of 9,10-phenanthrenequinone), and under anaerobic conditions.

Menadione (MQ) is a redox cycling compound with the empirical formula C_6_H_4_(CO)_2_C_2_H(CH_3_) and it is an analogue of 1,4-naphthoquinone having an additional methyl group. Also, it is known as a vitamin K_3_. Menadione is a pro-oxidant generating superoxide anion radicals. *Rhizobia* exposed to menadione responds by inactivation of free anion radicals generated by this exposure [36]. In this research, a low concentration of menadione acted as a stressor; therefore, it did not kill the bacteria but strengthened their resistance and increased their charge-transfer efficiency after the adaptation period. At ion concentrations below 1 mM, the zeta potential was independent of ionic strength (Figure 2A), and the bacteria displayed minor variation in their zeta potential in the presence of menadione; this effect was not dependent on the duration of incubation. When the ion concentration exceeded 1 mM, then the zeta potential of all samples increased along with the increasing ionic strength. No meaningful difference in zeta potential was observed between the presence or absence of various mediators and the duration of incubation. The influence of pH on the zeta potential of *Rhizobium anhuiense* was assessed. Considering that the natural living environment of the bacteria is ~pH 7.0, and due to metabolic processes and secretion of various metabolites the pH is often changed to the acidic side, further investigations in the pH range between 5.0 and 8.0 were performed. The research revealed that these pH alterations have not significantly influenced the zeta potential of the bacteria. The zeta potential remained negative over a wide pH range (Figure 2B). This reveals that the variation of pH does not influence electrostatic interactions between the bacteria and anode. The influence of the cultivation medium on the zeta potential of bacteria was also evaluated (Figure 2C). A Norris medium, pH 7.0, also positively influenced the zeta potential. The average zeta potential value of bacteria increased to −25 mV compared to the average zeta potential value of bacteria, which is −60 mV in PBS-based solutions with low ionic strength. This phenomenon is most likely due to the presence of different dissolved ions in PBS and Norris medium.

Bacterial cells with inherent negative charges are more favorable to adhere and subsequently immobilize to the positively charged electrode due to the electrostatic attraction. In this research, we observed a highly negative zeta potential in solutions with low ionic strength. Observation suggests that *Rhizobium anhuiense* possesses the inherent ability to adhere strongly to the surface of the anode.

**Figure 3:**
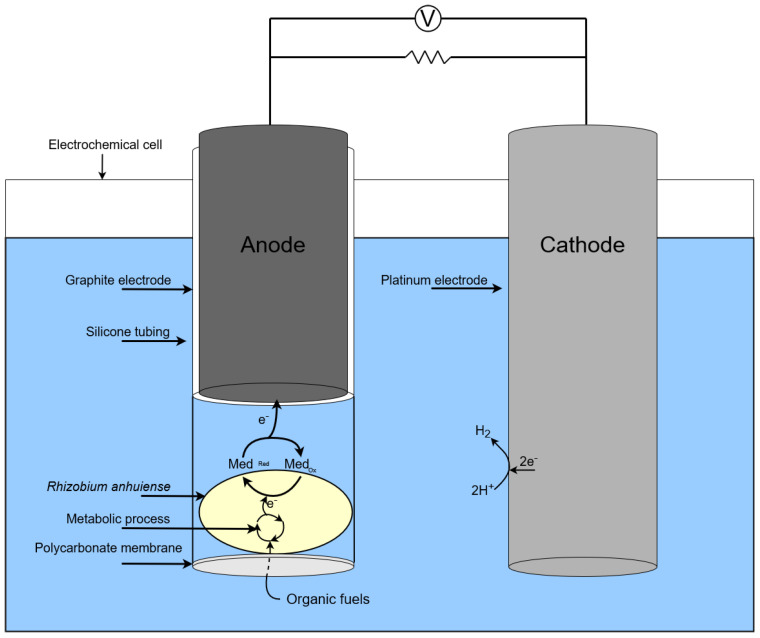
Scheme of a single chamber MFC based on *Rhizobium anhuiense*.

The action of single chamber MFC based on *Rhizobium anhuiense* is presented in the scheme in Figure 3.

Open circuit potential (OCP) was determined at loads on external circuits, and power density was calculated using these measurement results.

**Figure 4:**
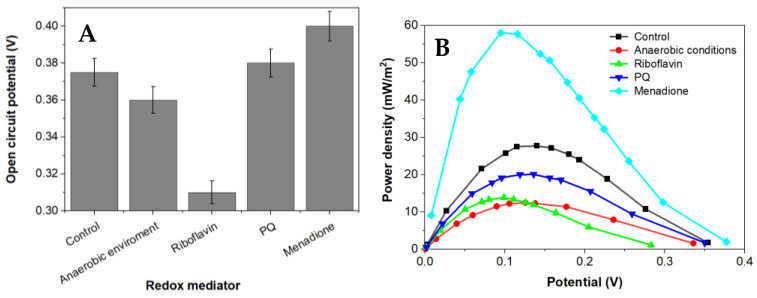
The dependence of capabilities to generate an electrochemical potential of *Rhizobium anhuiense* in 0.1 mM PBS, pH 7.0, the presence of several redox mediators (5 mM of menadione, 0.2 μM of riboflavin, and 4 mM of 9,10-phenanthrenequinone), and under anaerobic conditions. (**A**) the OCP values in presence of different redox mediators and (**B**) the dependence of power density on the potential value.

**Figure 5:**
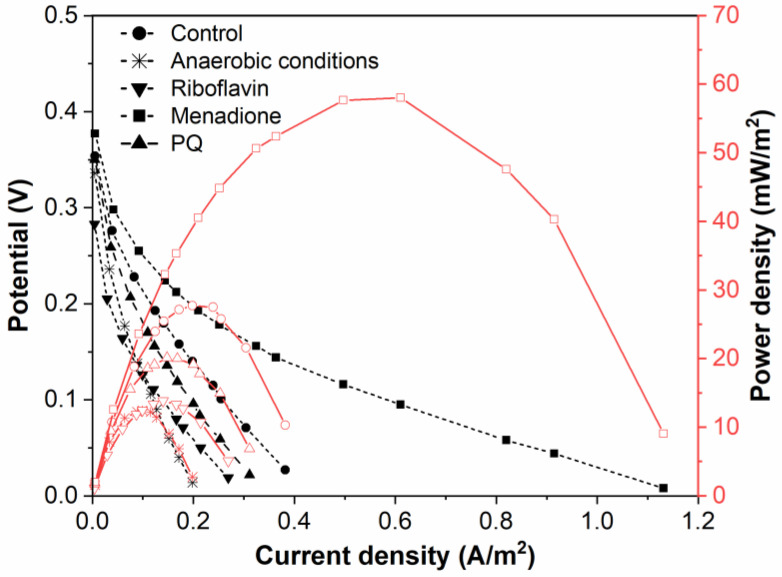
Polarization and power density curves of *Rhizobium anhuiense*-based BFC in 0.1 mM PBS, pH 7.0, in the presence of several redox mediators (5 mM of menadione, 0.2 μM of riboflavin, and 4 mM of 9,10-phenanthrenequinone) and at anaerobic conditions.

Power density curves and polarization were gathered for BFC based on *Rhizobium anhuiense* bacteria, which were treated with several redox mediators in different environments (Figure 4 and Figure 5).

Menadione is serving as an organic hydrophobic redox mediator enhancing charge transfer [28,29]. The efficiency of redox mediators is heavily influenced by the oxidation and reduction potentials of the used redox mediator. Redox mediators with a higher redox potential will more efficiently capture electrons from electron donors; however, electron transfer from redox mediators, which are characterized by a very high redox potential, to the charge transfer chain is not very efficient [39]. Electrical potential and power density of this designed MFC is shown in Figure 5. Riboflavin is known as an endogenous redox mediator, facilitating electron transfer rates, which promote the use of less of the generated power. Functional devices that have their energy supplied by fuel cells need to operate in conditions up to or equal to the power density maximum to function at high efficiency. Electrons transferred during this process are directly associated with the chemical reactions catalyzed by enzymes involved in the metabolic processes of microorganisms. Bacteria-based fuel cells are characterized by nonlinear power density. This nonlinearity can be used for efficient energy savings while being adapted to activate performance for a certain set of characteristics. A larger overall power density at a higher potential is a goal during the development of such systems because the maximum value of power density enables the generation of the greatest amount of electric current.

Application of a natural redox mediator—menadione—in MFC design enables an increase in both the voltage and power of the designed MFC, which also increases their applicability. In contrast, no positive effect was observed when riboflavin was applied instead of menadione. This effect can be related to the different solubilities of both these natural redox mediators, because menadione is soluble in a hydrophobic environment and enters the phospholipid membrane, whereas riboflavin is water-soluble and can be dissolved within the cell as well as within the extracellular environment. However, riboflavin is not well suited to shoot electrons through the cell membrane. Therefore, we can propose that menadione can more efficiently shuttle charge through the phospholipid membrane towards the electrodes in comparison to the charge transfer ability of riboflavin (Figure 5).

It is known that *Rhizobium anhuiense* can function in both anaerobic and aerobic conditions. Therefore, the ability of *Rhizobium anhuiense* to generate power was additionally tested in anaerobic conditions by the protrusion and saturation of the system with nitrogen gas. However, under anaerobic conditions, a significant decrease in all power generation-related characteristics was determined (Figure 1 and Figure 5).

A comparison of different biofuel cells is presented in Table 1.

It should be noted that the current density generated by this reported MFC, like that of most MFCs, is not particularly high; thus, to increase the density of the current, 3D electrode materials [40] and/or conducting polymer-based structures [1] can be used, allowing the magnitude of the current to be increased by several orders of magnitude [38].

**Table 1:** Comparison of different biofuel cells.

Source of Bacteria and Comments	Electrodes	Connection	Power or Power Density	Boosted Voltage	Ref.
Diluted swine wastewater as the substrate in the anode	Graphite felt/graphite felt	Multiple connection	-	Up to 6.6 V	[8]
Activated sewage sludge	Carbon fiber veil/carbon cloth	Multiple connection	770 μW	Up to 2.35 V	[10]
Glucose oxidase from *Penicillium funiculosum* 46.1 and horseradish peroxidase-based BFC	Graphite rod electrode/graphite rod electrode	Single	4.2 μW/cm^2^	530 mV	[12]
*Saccharomyces cerevisiae* with multi-walled carbon nanotubes (MWCNT) and 9,10-phenantrenequinone	Graphite rod electrode/platinum-based electrode	Single	113 nW/cm^2^	24 mV	[13]
*Saccharomyces cerevisiae* with 9,10-phenantrenequinone	Graphite rod electrode/platinum-based electrode	Single	22.2 mW/m^2^	178 mV	[14]
*Geobacter sulfurreducens*	Graphite/graphite	Single	-	0.47 V	[23]
*Saccharomyces cerevisiae* in the presence of methylene blue	Graphite/graphite with a proton exchange membrane (PEM) to separate the electrode compartments	Single	12.3 μW	250 mV	[26]
Thermophilic anaerobic digester used to treat wastewater from a brewery	Graphite felt/graphite felt	Single	1030 ± 340 mW/m^2^	-	[30]
*Rhizobium anhuiense* in the presence of potassium ferricyanide	Carbon felt (CF)/carbon felt (CF) with a Nafion 115 membrane to separate the electrode compartments	Single	1.07 mW/m^2^	245 mV	[35]
*Saccharomyces cerevisiae* in the presence of 2-methyl-1,4-naphthoquinone as a redox mediator	Graphite/graphite electrodes with a polycarbonate membrane to separate the electrode compartments	Single	0.408 mW/m^2^	24 mV	[41]
*Clostridium pasteurianum* in the presence of potassium ferricyanide	Carbon paper electrodes with proton exchange membranes (PEM) to separate the electrode compartments	Single	181.48 mW/m^3^	426 mV	[42]
*Saccharomyces cerevisiae* and polypyrrole composites in the presence of 9,10-phenantrenequinone	Graphite/graphite electrodes with a polycarbonate membrane to separate the electrode compartments	Single	47.12 mW/m^2^	390 mV	[43]
*Rhizobium anhuiense* in the presence of menadione, riboflavin, or 9,10-phenanthrenequinone as redox mediators	Graphite/platinum wire with a polycarbonate membrane to separate the electrode compartments	Single	5.5 μW/cm^2^	0.35 mV	This study

## 4. Conclusions and Future Developments

The development of microbial fuel cells based on electro-catalytic processes is among the novel topics that have recently emerged in the sustainable development of energy. However, not all microorganisms involved in metabolic/catalytic processes generate sufficient redox potential and/or are able to transfer electrons to electrodes. Therefore, in this research, we have assessed the applicability of microorganisms like *Rhizobium anhuiense* for microbial fuel cell design. In order to improve the charge transfer, we have tested several redox mediators, namely menadione, riboflavin, and 9,10-phenanthrenequinone. The best performance was determined for a *Rhizobium anhuiense*-based bioanode mediated by menadione with a 0.385 mV open circuit potential and 5.5 μW/cm^2^ maximal power density at 0.35 mV, which generated 50 μA/cm^2^ anode current at the same potential. These results indicate that the efficiency of the extracellular electron transfer is still not very high, and, therefore, the high internal resistance of some parts (e.g., the cell membrane) of the device remains one of the most significant challenges in the design of these MFCs. To increase the density of the generated current, 3D electrode materials and/or conducting polymer-based structures can be applied. It should also be noted that the optimal performance of extracellular electron transfer and the internal resistance of some parts of the electrochemical system remain two of the most significant challenges in the design of MFCs. Therefore, the improvement of charge transfer capabilities of the microorganism’s electrical capabilities would be another good target for boosting MFC performance and it can be applied to increase the density of current-generated 3D electrode materials and/or conducting polymer-based structures.

## Data Availability

Not applicable.
